# Djeen (Database for Joomla!’s Extensible Engine): a research information management system for flexible multi-technology project administration

**DOI:** 10.1186/1756-0500-6-223

**Published:** 2013-06-06

**Authors:** Olivier Stahl, Hugo Duvergey, Arnaud Guille, Fanny Blondin, Alexandre Del Vecchio, Pascal Finetti, Samuel Granjeaud, Oana Vigy, Ghislain Bidaut

**Affiliations:** 1 Aix-Marseille Univ, Marseille, F-13284, France; 2 Inserm U1068, Centre de Recherche en Cancérologie de Marseille, Marseille F-13009, France; 3 Institut Paoli-Calmettes, Marseille, F-13009, France; 4 CNRS UMR7258, Centre de Recherche en Cancérologie de Marseille, Marseille, F-13009, France; 5 Plate-forme de Protéomique Fonctionnelle, c/o Institut de Génomique Fonctionnelle, Montpellier, F-34000, France; 6 CNRS, UMR-5203, Institut de Génomique Fonctionnelle, Montpellier, F-34094, France; 7 INSERM, U661, Montpellier, F-34094, France; 8 Universités de Montpellier 1 & 2, UMR-5203, Montpellier, F-34094, France

**Keywords:** Database, CMS, Minimum Information, RIMS

## Abstract

**Background:**

With the advance of post-genomic technologies, the need for tools to manage large scale data in biology becomes more pressing. This involves annotating and storing data securely, as well as granting permissions flexibly with several technologies (all array types, flow cytometry, proteomics) for collaborative work and data sharing. This task is not easily achieved with most systems available today.

**Findings:**

We developed Djeen (Database for Joomla!’s Extensible Engine), a new Research Information Management System (RIMS) for collaborative projects. Djeen is a user-friendly application, designed to streamline data storage and annotation collaboratively. Its database model, kept simple, is compliant with most technologies and allows storing and managing of heterogeneous data with the same system. Advanced permissions are managed through different roles. Templates allow Minimum Information (MI) compliance.

**Conclusion:**

Djeen allows managing project associated with heterogeneous data types while enforcing annotation integrity and minimum information. Projects are managed within a hierarchy and user permissions are finely-grained for each project, user and group.

Djeen Component source code (version 1.5.1) and installation documentation are available under CeCILL license from http://sourceforge.net/projects/djeen/files and supplementary material.

## Findings

### Background

Advent of post-genomic era has seen a massive adoption of technologies that fundamentally changed the way biological assays are designed, data generated and collected, and further analyzed. Typical biological/genomic projects are now involving geographically spread laboratories that conduct large scale experiments that need to be analyzed in an integrated way [1]. Data must therefore be thoroughly annotated and mutually shared with fine-grained user permissions. It is also crucial that data annotation meet Minimum Information (MI) as well as standards defined in the lab where these data were generated. MI standards have been initially defined for microarray experiments (MIAME) [2] and have since been extended to other technologies, including flow cytometry (MIFlowCyt) [3] and proteomics (MIAPE) [4].

Several Laboratory Information Management Systems (LIMS) have been created over the years. Even though these have proven usefulness in instruments everyday usage, increasing data integration needs have underlined typical LIMS drawbacks in most laboratories.

Firstly, LIMS are usually conceived to manage data issued from a single technology type, such as DNA microarray [5,6], proteomics [7], high throughput sequencing [8]. This complicates further data integration and analysis since separate data types are typically stored on separate LIMS.

Secondly, any data evolution or reorganization implies reengineering and adopting a new LIMS. Most LIMS rather meet lab requirements at a given instant without taking into account the fact that technology, data formats, structures and devices are constantly changing and evolving, which restrains long-term use of the system.

Finally, each additional device installed in the laboratory implies training users on new laboratory practices since all LIMS are designed with different user interfaces (UIs). Subsequent fragmentation of systems and resources has a negative impact on quality control (QC). Moreover, administrating heterogeneous LIMS is a complex task that cannot be assumed by a typical wet laboratory structure that does not have the bioinformatics expertise and development resources to adapt tools to its specific needs. Because of these limits, typical LIMS do not favor interdisciplinary and translational collaborations and thus prevent the transfer of knowledge among laboratories generating heterogeneous information that has to be integrated.

In this report, we describe the Database for Joomla!’s Extensible Engine (Djeen), a new generation of LIMS that features a simple technological conception coupled with an advanced capacity of adaptation to be directly used or easily adapted to different technologies. The Djeen database scheme is kept simple to maximize generalization and application possibilities. Only data organization and metadata (annotations) are stored within a database, while experimental data is organized in the file system. In addition, the program is adaptable to any technology since no technology-dependent semantics were used in the code. Djeen can therefore evolve with user and laboratory needs. Since most technology can be managed with Djeen, this allows for unification of all information systems within the lab.

Djeen features a large functionality set (import–export, multi-technological implementation, advanced user/groups permissions) and is conceptually more advanced than other LIMS publicly available. Djeen qualifies as a Research Information Management System [9]. As such, Djeen addresses four fundamental issues in high throughput biomedical data management: data organization, data sharing, collaboration and publication.

Data organization consists in allowing straightforward data retrieval without the presence of the person who generated them. Therefore, data must be securely stored in a standardized way.

Data sharing consists in allowing several collaborators to access the same data, while avoiding data duplication or data reformatting. Collaboration is an important aspect of dataset entry and development; it allows several collaborators to manage and maintain the same data, which means that the data is not organized by a single individual. Finally, data publication encompasses secure data sharing for a large group of users (over the Internet or intranet).

### Related work

Many LIMS have been developed by large companies to respond to precise specifications responding to needs by a given laboratories. There exists also generic commercial LIMS that can be customized to specific needs. Some of them were confronted to a real situation in a large cell production facility [10]. In this paper, all facility members compared several LIMS according to multiple criteria and adopted a commercial LIMS. A consultant was hired for one year to adapt the LIMS to their specific needs, and adapt other modules, which implies significant means. On the contrary, Djeen was conceived to be used by small research groups with no or little dedicated bioinformatics support and few resources.

A commercial LIMS is hence not a ‘ready to use’ program and necessitate a very complex installation specific to end-user usage. Also, there is a strong dependence on the software’s vendor and his ability to follow up on the product and insure longevity, which is not guaranteed. Djeen being open source and under CeCILL license, anyone can contribute to it and it will stay available.

A number of publicly available open source LIMS exist, and partially addressed the issues described in this paper. Many LIMS are inherently limited to a single technology type, i.e. Proteomics (ms_lims, [7]) or microarray (BASE [6], or the Longhorn Array Database [5]), which is a serious limitation for many laboratories, who need the same system to store all their data. Djeen allows for managing heterogeneous datasets within the same system.

Other LIMS can be used to manage multiple technologies. MADMAX [11] allows for the storage and analysis of heterogeneous genomics data in the same repository. However, its implementation is rather complex and based on an Oracle (Redwood City, CA, USA) Database Management System (DBMS), which can be a limiting factor for some laboratories with limited resources. Djeen is based solely on open-source technology, being its database (MySQL or PostgreSQL) or the Content Management System (Joomla!). SIGLa (Systema Integrado de Gerência de Laboratoriós, [12]) is based on a workflow management tool that makes it also adaptable to multiple usages. The concept of workflows is not needed by all users and increases dramatically the complexity of data entry. Therefore, workflows are not hard-coded into Djeen to avoid technology-specific code but can be used with annotations when needed.

BonsaiLIMS [13] is a lightweight LIMS developed initially to centralize patient records for translational research. However, BonsaiLIMS features only a very limited model and functionality set as compared to Djeen, which works “out of the box” after a deployment from the Joomla!’s component deployment interface. Djeen features more functionality, such as templates group permission management and data export.

### Implementation

Djeen is a Research Information Management System developed to meet the four type of functionalities described above:

Data organization

Djeen structures data into a hierarchical system allowing creating and managing complex data structures hierarchically with high flexibility while enforcing standardization and Minimum information standards. Djeen is able to manage projects containing heterogeneous data types while maintaining homogeneous annotations through the optional use of templates.

Data sharing

Data is shared through fine-grained user permissions to strengthen data security. Several user roles are defined and granted with gradually increasing permissions. This allows managing data that is completely confidential as well as sharing it with specified users and groups, or release it publicly over the Internet.

User collaboration

User collaboration goes beyond data sharing. It implies that datasets are built and contributed to by several collaborators while general project structure and annotations are under the control of a project owner. In Djeen, once permissions are set to appropriate users or groups, project administrators can control homogeneity of annotations with templates. Templates allow enforcing data integrity and homogeneity as well as quality control (QC). Their role is detailed in the ‘Data organization and Djeen elements’ section.

Publication

To be able to publish datasets and meet needs for data sharing and collaborations, Djeen was developed as a Web application.

Djeen software administration

A fifth item was taken into account when designing Djeen. Djeen was conceived as a Web application that is simple to install, easy to learn and to administrate. A Djeen instance is maintainable by a research group with no or little dedicated bioinformatics human resources, and usable with a minimal training.

#### Content management system

Djeen was developed as an extension of the Joomla! Content Management System (CMS).

CMS are a category of applications allowing the management and the publication of content, “content” being defined rather loosely (see [14] for a review of CMS usage in bioinformatics). In our case, it is related to scientific information, and more precisely, data and their annotations. CMS are very attractive since they are characterized by a modular design and allow for development of new applications for visualizing content. Reuse of CMS tools for web application development potentially simplifies things and allows programmers to focus on the application features instead of spending time on redeveloping a number of generic functionalities, such as site administration and visual design.

As compared to a home grown development, developing a web application as a CMS extension allowed us to maximize the security by taking advantage of the existing authentication system. Additionally, security does not depend entirely on the application itself but also on the local network configuration.

The appropriate choice for a CMS is crucial especially considering security issues [14]. We carefully choose the CMS on several criteria, including active support, presence of a documented API, quality of the interface in terms of look and feel, and simplicity of administration and installation. In regard to all these considerations, we decided to develop Djeen as a Joomla! 1.5 component.

Joomla! version 1.5 is an open-source Content Management System (http://www.joomla.org) featuring a documented API to create advanced extensions based on a Model-View-Controller (MVC) Design Pattern [15]. Additionally, it is backed-up by a large community of developers that quickly releases security patches and new versions and update documentation.

#### Integration within the Joomla! CMS

**Figure 1:**
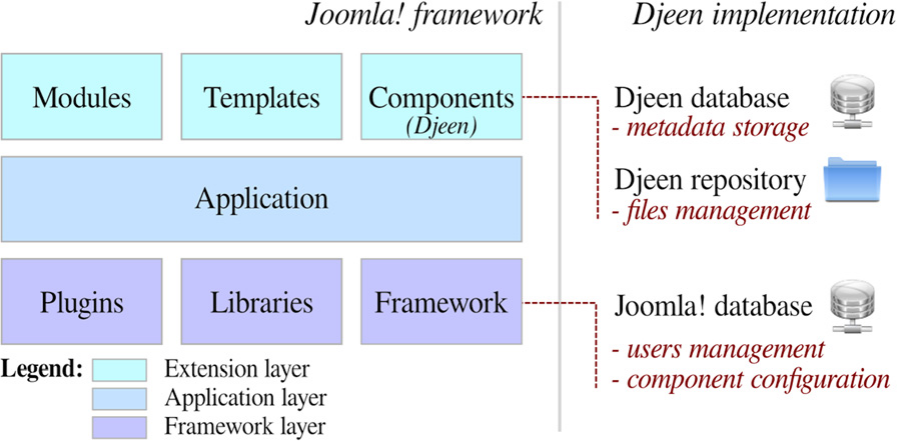
**Joomla! API and Djeen implementation. **This figure shows the Joomla's API and the mechanisms of interaction between Djeen and Joomla!. The right part of the figure represents the three tiered Joomla! API (adapted from Joomla's documentation, (http://docs.joomla.org/Framework/1.5#Packages_and_Classes). This architecture defines the software from data access to the final application display through a Model-View-Controller (MVC) model. The left part of the figure shows specific Djeen main components (Djeen database, file repository) as well as Joomla!'s components (Joomla!'s database, containing user information and component configuration) reused by Djeen. Djeen uses the framework layer to interact with Joomla! and manages users’ connections and the application layer for display.

To understand how Djeen interacts with Joomla!, a summary of its architecture and function is given. As shown in Figure 1, Joomla! was conceived as a three tier system based on this MVC structure, to separate data access (Models), rendering system (Views) and actions (Controllers).

The purpose of the framework layer is to manage data within Joomla!, it includes different classes and libraries to access database and file system. The application layer manages Joomla!'s UI, such as administration panel and public website. Finally, the extension layer includes templates to change the website rendering and extensions to change or extend Joomla!'s functionalities. Modules are used to display additional data bits and pieces into specific ‘boxes’. Components are a more advanced type of extensions that allow developing full fledged applications while granted access to the Joomla! API. Djeen is a Component and implements its own database and a dedicated repository.

By using the Joomla! API, Djeen is deployed and managed from the Joomla! administrator panel. No third party software or client is needed and compatibility was validated on the Mozilla Firefox^®^ and Google Chrome^®^ browsers (current versions as of publication). The application is embedded to the Joomla! front-end website through the application layer.

Data security is ensured by a complete separation of Djeen and Joomla! databases to facilitate backup and keeping Djeen as an independent component at every level. Djeen objects are stored within a specific relational database (PostgreSQL and MySQL drivers are provided) while files are simply stored in the file system. The connection to the Djeen external database is ensured by a second security level that uses an encrypted password stored into the component configuration. Several databases can be maintained, and each instance has its own password.

#### Data organization and Djeen elements

In this section, Djeen objects (such as Projects and Templates) are refereed to with a capital letter. These are simply referred to as generic terms in subsequent sections.

**Figure 2:**
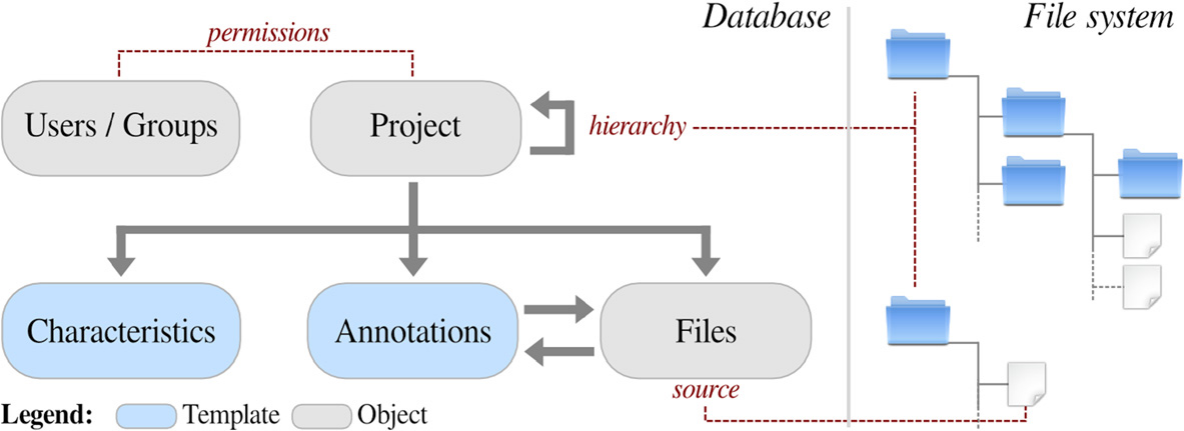
**Djeen data organization model. **Djeen separates Projects hierarchy, Templates and Annotations on one side (these are stored in the Djeen relational database) and the data itself (stored in the file system). Data stored into the database are divided into several objects, each of them being represented by a database table, linked to a class model in the MVC architecture. Project is the primary Djeen organizational element. It allows the construction of a hierarchical tree of sub-Projects and includes metadata about itself and stored files. The File object is the most basic and central piece of information within Djeen, since it represents the lowest granularity level in most high-throughput analyses. This project/files hierarchical tree is structurally mirrored into the file system. Metadata are divided into Characteristics (related to Projects) and Annotations (related to Files). Those two types of metadata can be saved into Templates, allowing users to reuse them into other Projects. The Users object is a specific table containing user and group information. It supersedes the Joomla! system by formation specific to Djeen that allows managing specific permissions and groups while permitting reuse of all the features already implemented in the CMS, such as authentication or e-mailing.

Figure 2 shows the Djeen data model main elements and the duality files-database. The File object is the central piece of information within Djeen, since it represents the lowest granularity level in most high-throughput analyses. This model and the associated code were designed to be technology-independent and free of specific semantics to stay adaptable to multiple data types and as generalizable as possible. In the database side, the main Djeen entity to manage data is the Project. Projects are organized in a hierarchy and a Project can reference multiple sub-Projects. This hierarchy is mirrored in the corresponding directory organization on the file system side. Projects are linked to Files issued from experimentation or analysis using this directory organization. Projects are equivalent to folders and files are simply stored within these folders. This organization is very intuitive and simple to manage and backup.

Files and Projects are annotated by two types of metadata. The Annotations are related to Files and the Characteristics are specifically related to Projects. Annotations contain sample or condition-specific information while Characteristics contain general Project information and descriptions.

To allow reusing metadata for similar Projects, Templates were introduced. They allow storing a predefined list of Annotations and Characteristics that will be used when creating a Project. Templates have specific owners and cannot be modified once they are used in a Project to prevent further unwanted modifications.

No constrains are imposed by Djeen implementation on Project structure or Annotations types. This allows for the use of multiple data types in the same management system. However, Projects manager can optionally use Templates to enforce the type of Annotations to be provided by the experimentalist and enforce MI compliance if necessary. Data provenance, for instance, can be encoded as a mandatory annotation field. Templates also allow structuring the Project, to permit further data integration and promote QC by being consistent with MI and sample annotations. In addition to Templates and Annotations, Djeen allows highlighting (with a flag) a specific annotation that corresponds to the biological question asked in a Project.

Djeen allows data sharing under an advanced permission system that supersedes the native user management system of Joomla! by adding specific information. Four roles have been defined (Superadministrator, Administrator, Moderator, and User) granted with specific permissions on data, User/Groups and Template management. A given Project is owned by an Administrator or a Moderator who has the role of Project owner. He controls annotations and user permissions on files. Projects and sub-Projects permissions can be granted to specific user groups to finely manage access of large scale collaborative projects.

#### Web Interface and Djeen functionality

Djeen’s UI was designed to facilitate navigation and manipulation of data as intuitively as possible and features a fast learning curve. To optimize interoperability with Joomla! and maximize its maintainability, Djeen was mainly developed in PHP. A data uploader was developed in Perl to allow massive data transfers (of several GBs) for importation of large sets of raw data. All table rendering and user interaction code was implemented an Ajax/Javascript layer through the Mootools library (http://mootools.net). Additionally, a customizable dialog box was made available for administrators to display information such as an End-User License Agreement (EULA) for users, as well as any other information or error messages.

**Figure 3:**
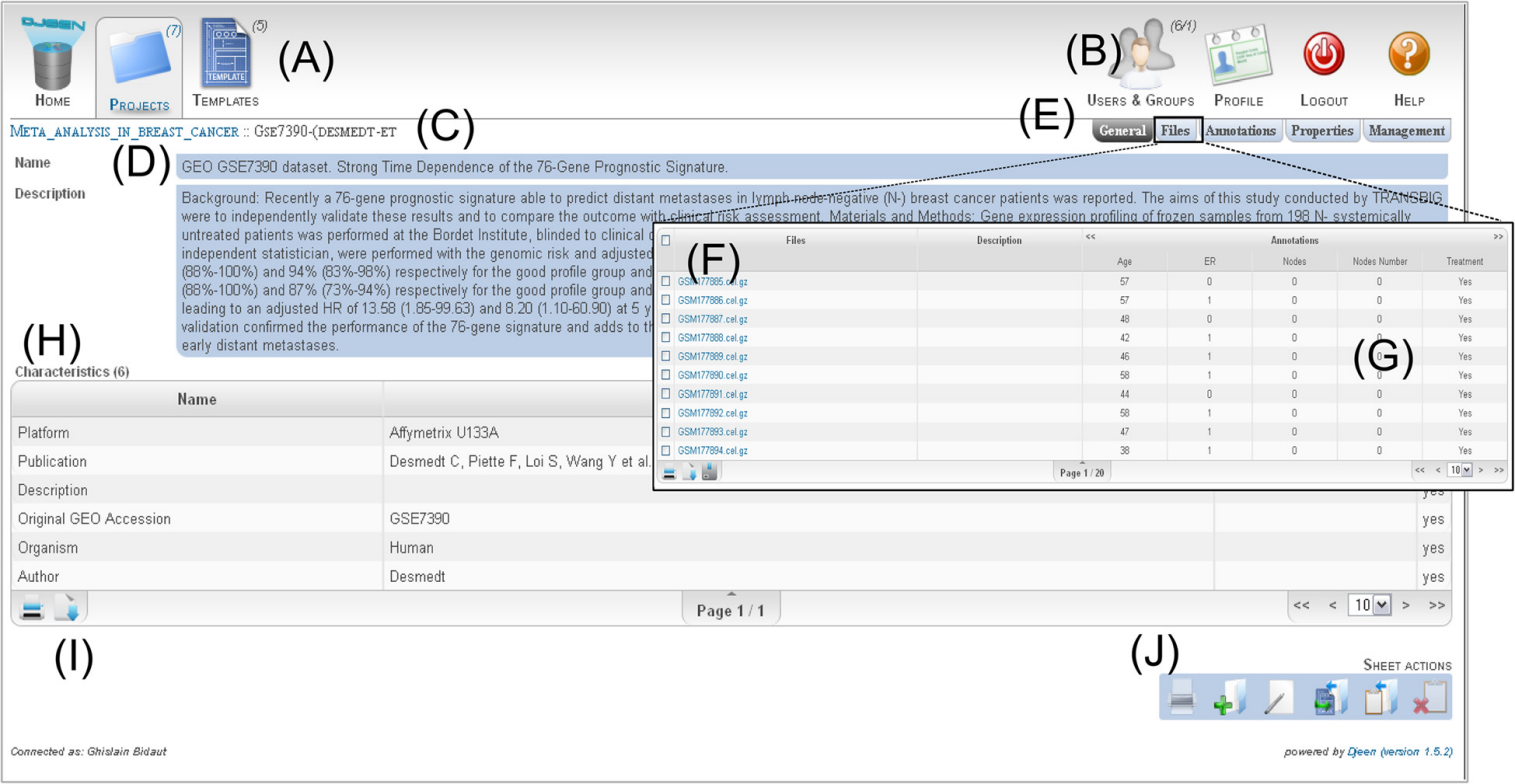
**Djeen Web Interface. **This figure shows the Djeen web interface opened on the project view, as seen as connected as a user with read permissions. The web interface is embedded within Joomla! (Not represented here), and presents all elements related to this particular view as well as some elements common to several views. Among the common element to all Djeen views are the main icons (A). These allow navigation between the main views, which are: User home view, projects view and templates view. Icons in B, also accessible from all views, are related to administrative tasks, namely “User and groups management”, current user “Profile” management, “Connection” icon, and access to “Help”. Breadcrumb (C) allows locating the current project in the global hierarchy. In D are the general element to identify the project, “Name” and “Description”. E contains sub view of the project, which are the “General” view (the one currently displayed), “Files” (represented in the F view overlaid on the figure), “Annotations”, which contain the details of each annotations displayed in G. “Properties” and “Management” allow the management of user permissions and other technicalities (project acronym, template identifier). The (G) table in the File view shows the annotation values or each sample. Table H lists the project characteristics: In (I) are the “Print” and “Export” icons, which are common to each table. Actions (J) icons allow global action on the current project, such as “Print”, “New Project”, “Edit”, “Copy project as Template”, “Copy into the Clipboard”, and “Clear Clipboard”.

The Djeen UI main elements are represented Figure 3. Main icons (A) allow for quick access to projects and experiments and search dialog. Other icons (B) allow for template management, user and group management, current user options, and connection to the system. The breadcrumbs (C) allow for navigating project hierarchy. Panel (D) shows the main project view. It features general description, owner and creation date, template used and characteristics table (H). The tabs (E) allow for management of sub-projects, files, annotations, project history and specific user permissions. The “Files” tab opens a panel (overlaid here) displaying the list of files linked to the current project (F), annotated using the annotations table (G). Moreover, users can generate snapshots of the whole project by using the “History” function in (E). Such a snapshot allows project manager to generate a data freeze for publication of a project. A notification zone displays messages returned by the system (I) in an elegant and non-intrusive manner. (I) icons are functions present in most tables, namely “Print” and “Export”. Icons (J) are general project actions including “Print”, “New Project”, “Edit”, “Copy as template”, “Copy in Clipboard” and “Clear Clipboard”.

Annotations can be either typed within the Djeen interface or imported from preformatted CSV files. Direct annotation entry is facilitated with an integrated advanced table formatting tool. Multiple data files can also be imported at once (using zip or tar archives) and default annotation values specified to speed up data entry. In a set of annotations, the variables that represent the biological question can be flagged to explicitly mark the datasets that could potentially be integrated since they address similar biological question.

### Results and Discussion

To demonstrate Djeen functionalities and purpose, we describe a detailed microarray importation and management procedure for a microarray dataset of breast cancer patients annotated with clinical conditions. While this specific example is concerned with microarrays, it is also easily applicable to other data types. Specific tutorials detailing management of microarray and flow cytometry data are accessible from the Djeen documentation. The dataset used in the current example [16] is publicly available from the Gene Expression Omnibus (NCBI GEO, http://www.ncbi.nlm.nih.gov/geo) under the accession number “GSE7390” This dataset spans 198 CEL files, each of them corresponding to a single breast cancer tumor sample. To be able to enforce annotation format and keep consistency among projects, we set up a template for sample annotation that can be re-used by other projects. This project will be part of a hierarchy and defined as a sub-project of a general “Breast Cancer” project. This allows the future inclusion of other projects at the same level within Djeen.

### Preparing the project hierarchy within Djeen

First, the data must be stored at the appropriate location within Djeen. This depends on the local organization of data as well as on the studied data type. In all cases, careful consideration of the adopted hierarchy must be taken. For our own purpose, we will create a meta project “breast cancer” and then a sub project “Desmedt et al. Local Copy” for the data at hand. This is done by clicking on the general tab “Projects”. By using the add button (“+”), a new meta-project called “Meta_Breast_Cancer_Transcriptome” can be added. Dataset description can be filled here, for instance “Meta Breast Cancer Transcriptome project grouping multiple datasets”. Permissions can be specified to limit access to the data. Since this is a publicly available dataset, it can be shared with people already registered to the system. All changes are saved by clicking on the “Save” button.

Similarly, a sub-project is being defined to store and describe the data. This is done by clicking the “sub-project” tab, creating a new project called “GSE7390 (Desmedt et al.)”. No template is being defined at this point. Again, a description can be added as free text. Permissions are also left to their default settings: access to registered users. Again, changes are saved by a click on “Save”.

The previous steps generate the following project hierarchy:

“Meta_Breast_Cancer_Transcriptome'->'GSE7390”

### Importing microarray data (CELs files)

The full test dataset can be directly downloaded from the Gene Expression Omnibus database using the accession number GSE7390. After downloading the full archive, all CEL files are imported within Djeen by a click on the “File” Icon under the “GSE7390” project. Djeen has a built-in on-the-fly decompression capability for direct importing of tar, tar.gz and zip files.

After importation, all files are accessible from the “File” tab.

### Adding and formatting a MIAME-compliant transcriptome template

At this step, a MIAME-compliant template will be applied on the data. This template has two purposes. First, it allows a project administrator to insure that the data is MIAME compliant and second, it allows re-using the same annotations for other datasets.

Templates are added from the Template section of Djeen. For our example, it will be named “Breast Cancer Transcriptome Template”. Keywords can be specified as follows: “Transcriptome Breast Cancer Clinical Data”. The description can be specified as 'Template for describing microarray of breast cancer samples'. Changes are saved in the system by a click on “Save”.

Then, the two template key elements must be populated: Characteristics (variables specific to the dataset) and Annotations (variables specific to samples). In our case, the Characteristics are: “Author”, “Original GEO/ArrayExpress Accession Number”, “Organism”, “Platform Vendor” and “Platform Type”.

**Figure 4:**
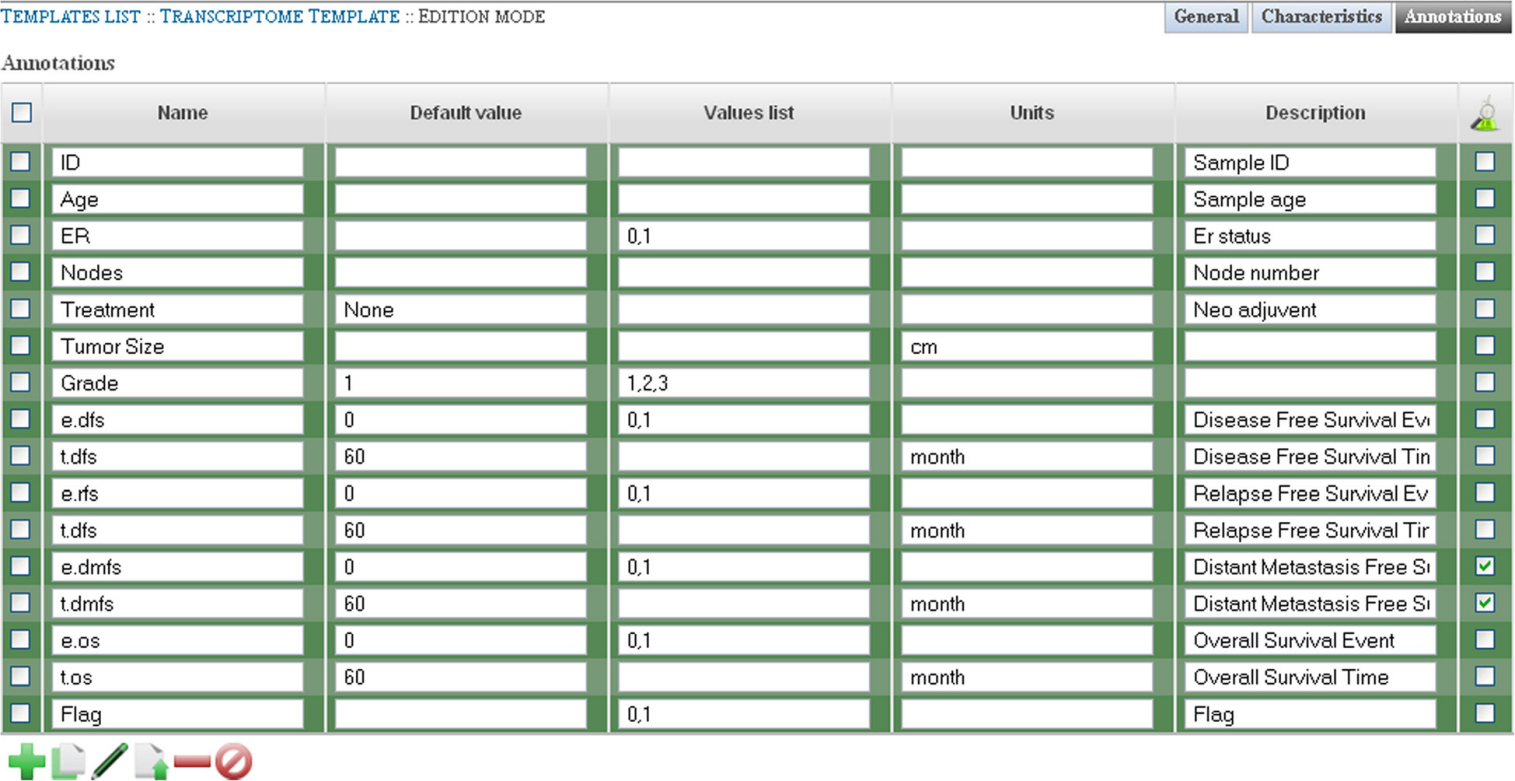
**Template Edition Interface. This figure shows the template edition view. **It allows for editing the list of annotations and their corresponding values for a template. Two mechanisms have been set up to control values that are allowed for a given annotation. If a default value is specified, this value will be set up by default when importing data if no other data is mentioned. Otherwise, a list of values can be specified, to limit the range of possible data. When importing data, the first value will be specified by default if no other data is specified. All metadata (characteristics and annotations) are edited with a similar interface.

Values list and description can be added if necessary. Similarly, Annotations are populated as specified Figure 4. “e.dmfs” and “t.dmfs” are marked as “Experimental Question”.

Next, the “Breast Cancer Transcriptome Template” must be linked to the “GSE7390 (Desmedt et al.)” project. This is done from the project view by editing the template field. Once done, 5 Characteristics and 6 Annotation(s) were added to the project.

The final step is to populate Characteristics and Annotations. The project contains 198 samples, making the process of manual annotation very lengthy. To ease the process, we wrote an annotation file that can be imported directly within Djeen (available from Djeen documentation page at http://sourceforge.net/projects/djeen/files). However, it has to be formatted to be imported, especially to fit the sample database Ids.

This is done by generating an empty annotation file by exporting current file annotation data using “Files”-> “Save the file to annotation.txt”, and editing “annotation.txt” with a spreadsheet and importing data from the “Desmedt-annotations.txt” file contained in the Affymetrix.zip archive.

This file can then be imported directly with Djeen by the ‘Import’ and ‘Save’ functions. The following message should appear: *198 file*(*s*) *correctly edited* - *3168 file annotation*(*s*) *updated*. At this point, the project has been correctly annotated. The annotation structure can be reused in other datasets if necessary.

The last step consists in setting up proper permissions to share datasets. Read and Edition parameters can be set from the Permission tab. Also, specific Read and Edition parameters can be set for user groups.

The project is now completely loaded within Djeen and can be further edited and shared. Data can then be downloaded from Djeen. Individual samples can be downloaded from the “File” tab while the whole dataset can be downloaded by performing a snapshot and downloading the resulting archive.

### Conclusion

Djeen is a new Research Information Management System designed to answer laboratories’s growing needs for flexibility in data management. It allows the sharing, secure storing and annotation of heterogeneous data types, streamlines repetitive annotations tasks and features import functions. Djeen organizes projects within a natural hierarchy similar to files and folders organization on disk. Templates (optionally used) allow automation and MI Information. These features were demonstrated in this report with the demonstration of a real use case for microarray data.

Djeen features a user-friendly installation procedure and web interface. It is conceived as a Joomla! component, has a fast learning curve and can be deployed over MySQL or PostgreSQL.

Future development include setting up an advanced interface for data requests, a scripting module to perform data processing directly from the UI, and an advanced authentication system using LDAP.

## Availability and requirements

• **Project name:** Database for Joomla!’s Extensible Engine

• **Project home page:** https://sourceforge.net/projects/djeen

• **Operating system(s):** Platform independent

• **Programming language:** PHP/Javascript/SQL/Perl

• **Other requirements:** A Linux server running Apache2/Perl 5.x//PHP 5.3+/MySQL or PostgreSQL

• **License:** CeCILL

• **Any restrictions to use by non-academics:** Please contact directly the authors for additional information.

## Abbreviations

CMS: Content Management System; EULA: End-user license Agreement; Djeen: Database for Joomla!’s Extensible Engine; RIMS: Research Information Management System; MI: Minimum Information; MIAME: Minimum Information About A Microarray Experiment; UI: User Interface.

## Competing interests

Authors declare that they have no competing interests.

## Authors’ contributions

OS designed the database scheme, software architecture and implemented the web interface and database. He also wrote the manuscript. HD developed installation procedure, finalized code for release and wrote documentation. AG designed import/export functions. FB and ADV contributed the UI (projects and file views). PF provided initial discussions in interface and microarray data management. SG and OV designed data and template models and provided helpful suggestions. GB funded the project, wrote documentation and finalized the manuscript. All authors participated to debug and release the 1.5 version. All authors read and approved the final manuscript.
